# Orchestral manoeuvres in the light: crosstalk needed for regulation of the *Chlamydomonas* carbon concentration mechanism

**DOI:** 10.1093/jxb/erab169

**Published:** 2021-04-20

**Authors:** Indu Santhanagopalan, Rachel Wong, Tanya Mathur, Howard Griffiths

**Affiliations:** Department of Plant Sciences, Downing Street, University of Cambridge, Cambridge, UK; Department of Plant Sciences, Downing Street, University of Cambridge, Cambridge, UK; Department of Plant Sciences, Downing Street, University of Cambridge, Cambridge, UK; Department of Plant Sciences, Downing Street, University of Cambridge, Cambridge, UK; University of Illinois, USA

**Keywords:** Carbon concentration mechanism (CCM), chaperones, *Chlamydomonas*, CIA5, photorespiration, photosynthesis, pyrenoid, retrograde signalling

## Abstract

The inducible carbon concentration mechanism (CCM) in *Chlamydomonas reinhardtii* has been well defined from a molecular and ultrastructural perspective. Inorganic carbon transport proteins, and strategically located carbonic anhydrases deliver CO_2_ within the chloroplast pyrenoid matrix where Rubisco is packaged. However, there is little understanding of the fundamental signalling and sensing processes leading to CCM induction. While external CO_2_ limitation has been believed to be the primary cue, the coupling between energetic supply and inorganic carbon demand through regulatory feedback from light harvesting and photorespiration signals could provide the original CCM trigger. Key questions regarding the integration of these processes are addressed in this review. We consider how the chloroplast functions as a crucible for photosynthesis, importing and integrating nuclear-encoded components from the cytoplasm, and sending retrograde signals to the nucleus to regulate CCM induction. We hypothesize that induction of the CCM is associated with retrograde signals associated with photorespiration and/or light stress. We have also examined the significance of common evolutionary pressures for origins of two co-regulated processes, namely the CCM and photorespiration, in addition to identifying genes of interest involved in transcription, protein folding, and regulatory processes which are needed to fully understand the processes leading to CCM induction.

## Teaser

The interplay of photosynthesis with carbon acquisition and fixation in *Chlamydomonas*, regulating its inducible carbon concentration mechanism that is dependent on cellular machineries of transcription, protein folding, and retrograde signalling, is reviewed here.

## Introduction

The carbon concentration mechanism (CCM) traits found in algae (and cyanobacteria) have evolved to improve the operating efficiency of Rubisco, which is normally packaged within a specific microcompartment: in algae, this is the chloroplast pyrenoid. Inorganic carbon, in the form of bicarbonate, is delivered to the chloroplast stroma using a series of membrane transporters. Saturating internal CO_2_ concentrations (Ci), ~40× above ambient (Badger *et al.*, 1980), are generated within the pyrenoid by strategically placed transporters of inorganic carbon and carbonic anhydrases (CAs) (Moroney and Ynalvez, 2007; Meyer and Griffiths, 2013; Hennacy and Jonikas, 2020). The availability of a sequenced genome (Merchant *et al.*, 2007), transcriptomic studies for synchronized cells across 24 h light/dark cycles (Zones *et al.*, 2015; Strenkert *et al.*, 2019), and extensive mutant libraries (Li *et al.*, 2016, 2019; Vilarrasa-Blasi *et al.*, 2020, Preprint) for *Chlamydomonas* have provided additional opportunities for CCM characterization.

Substantial molecular and mechanistic advances in our understanding of the algal CCM have been recently reviewed (Meyer and Griffiths, 2013; Meyer *et al.*, 2017; Goudet *et al.*, 2020; Hennacy and Jonikas, 2020). CCM induction is associated with enhancement of aggregation of Rubisco with specific linker proteins (Mackinder *et al.*, 2016; He *et al.*, 2020; Meyer *et al.*, 2020) in the pyrenoid surrounded by a starch sheath, with an existing network of knotted tubules making connections with thylakoid stacks (Engel *et al.*, 2015; Meyer *et al.*, 2017). Establishment of the CCM must be co-ordinated between the chloroplast and nucleus in sensing induction stimuli, triggering CCM gene expression, translation, and intracellular transport and assembly of CCM proteins.

The aim of this review is to characterize the various novel aspects of molecular mechanisms leading to mechanisms of CCM induction and establishment. We explore the regulatory interplay between environmental sensing, photosynthesis, and CCM induction that is critical for *Chlamydomonas* and identify future avenues for investigation. First, we consider the control of nuclear gene expression and the need to identify transcription factors (TFs) associated with sensing the different environmental stimuli—light and CO_2_; second, regulation of export of translated proteins and folding within the chloroplast, and associated chaperone systems; third, formation of the pyrenoid matrix, starch sheath, and intrapyrenoidal tubule network; fourth, the role of retrograde signalling in delivering signals to alter nuclear gene expression; and, finally, we discuss the potential evolution of CCM induction from existing photorespiration regulatory mechanisms, through the master regulator CIA5.

## CCM induction and control of gene expression

Changes in [CO_2_] in the external medium have traditionally been thought to be sensed through Ci of the photosynthesizing algal cell and conveyed to the nucleus to change the expression of genes that turn on/off the CCM. Studies carried out with asynchronous cells grown under continuous light revealed [CO_2_]-dependent expression for >5000 genes at the transcription level (Brueggeman *et al.*, 2012; Fang *et al.*, 2012). Over 600 of these differentially expressed genes identified in these genome-wide studies have been implicated in the CCM (Mackinder *et al.*, 2017). With several hundred genes orchestrating the CCM, the following questions come to the fore: (i) what are the regulators in the nucleus that respond to [CO_2_]/Ci changes, and (ii) how do they bring about changes at the transcriptional level? In this section, we discuss regulatory mechanisms operating at the transcriptional level to modulate the inducible CCM in *Chlamydomonas*.

### CIA5: the ‘master-regulator’ of the CCM

One of the first interesting candidates to be identified as a ‘CCM master regulator’ was *CIA5/CCM1*. *CIA5* was identified in 1989 as essential for growth in limiting CO_2_ conditions through studies on a UV-generated mutant, *cia5* (Moroney *et al.*, 1989). Studies in the early 2000s established *CIA5* as being essential for induction of expression of several CCM genes encoding inorganic carbon transporters, *HLA3* and *LCI1*; CAs, *CAH3* and *CAH1*; alanine α-ketoglutarate aminotransferase, *ATT1*; pyrenoid protein, *EPYC1*; a TF, *LCR1*; and mitochondrial membrane proteins, *CCP1* and *CCP2* (Fukuzawa *et al.*, 2001; Xiang *et al.*, 2001; Miura *et al.*, 2004). It must be noted that genes denoted as CCM genes in this review are based on previous findings (Mackinder *et al.*, 2017; Strenkert *et al.*, 2019). Genome-wide studies (Fang *et al.*, 2012) showed *CIA5-*dependent expression for 15% genes, but only around half of these *CIA5*-dependent genes responded to changes in [CO_2_]. Furthermore, the mechanism of CIA5 in helping cells acclimate to [CO_2_] changes is not understood. CIA5 is proposed to be a TF based on the presence of two Zn-finger domains (Fukuzawa *et al.*, 2001; Xiang *et al.*, 2001). The DNA-activating region (Chen, 2016) and the [CO_2_]-dependent domain that triggers the expression of CIA5-dependent CCM genes (Xiang *et al.*, 2001) lie in the C-terminal end sequences of 130 and 54 residues, respectively. Primarily, the expression of *CIA5* seems to be [CO_2_] independent (Fang *et al.*, 2012), and associated [CO_2_]-dependent expression changes in the genome are believed to be mediated by post-translational modifications of CIA5 (Chen, 2016). This is supported by CIA5 having several putative sites for phosphorylation, glycosylation, and myristoylation (Fukuzawa *et al.*, 2001), and anomalous electrophoretic mobility (Chen, 2016).

Absence of evidence for DNA–CIA5 complexes suggests that CIA5 might act indirectly through other proteins, although no such proteins have been identified (Kohinata *et al.*, 2008). Recombinantly expressed full-length CIA5 showed very weak affinity *in vitro* for the 9 bp sequence (GGGGCGGGG), identified from analysis of upstream sequences of select CIA5-dependent genes (Chen, 2016). However, no motif-dependent binding for CIA5 could be established *in vivo* when genes with upstream mutated motifs showed similar expression patterns to those of non-mutated motifs (Chen, 2016).

Understanding the CIA5 mechanism will require identification of the different post-translationally modified forms of CIA5 and the corresponding *cis*-regulatory elements of CCM genes. The roles played by CIA5 are revisited in the context of chaperone expression in ‘Chaperones and the import and assembly of chloroplastic CCM proteins’, and the implications of strikingly similar CIA5-dependent expression profiles of photorespiratory and CCM genes are explored below.

### Other transcriptional and post-transcriptional regulators of the CCM

A search for other CCM TFs and transcription regulators (TRs) has been made over the years. LCR1 (Yoshioka *et al.*, 2004) is a Myb TF that plays a crucial role in the CCM by regulating expression of *CAH1*, *LCI1*, and *LCI6*. *LCR1* expression is [CO_2_] and CIA5 dependent. Absence of *LCR1* leads to a reduction in affinity for Ci (Yoshioka *et al.*, 2004).

Sequence analysis identified 234 genes as potential TFs and TRs in *Chlamydomonas* (Riano-Pachon *et al.*, 2008), and they need to be checked for their activity in CCM regulation. A recent proteomic analysis (Arias *et al.*, 2020) on nuclei obtained from *Chlamydomonas* grown in 5% CO_2_/0.04% CO_2_ revealed the presence of 117 proteins which were potential TFs/TRs. Of these 117 nuclear proteins, 35 were of differential abundance dependent on [CO_2_], and these are listed in Table 1. It is worth noting that neither CIA5 nor LCR1 was detected in the nuclear proteome in this study. However, the candidates identified in this study might act as a good starting point for investigating other TFs and TRs regulating the CCM.

**Table 1.** Transcription factors and regulators occurring with different relative abundances in *Chlamydomonas* grown in low (0.04%) and high (5%) CO_2_ conditions (Arias *et al.*, 2020)

Protein	Description	TF family^*a*^	Fold change
**Transcription factors**			
Cre01.g000050.t1.1	RWP-RK Transcription Factor	RWP-RK	5.5
Cre10.g444450.t1.1	Predicted Protein	C3H	3.1
Cre14.g625802.t1.1	Ring Finger Protein-Related	FHA	2.9
Cre16.g656250.t1.1	U1 Small Nuclear Ribonucleoprotein	CSD	2.5
Cre17.g714500.t1.2	Histone H2A	CCAAT	2.5
Cre06.g288750.t1.2	Nuclear Rna Cap-Binding Protein	CSD	2.4
Cre06.g254650.t1.2	Zinc Finger Protein 183	C3H	1.9
Cre14.g632050.t1.2	RPGR-Interacting Protein 1 Related	VARL	1.9
Cre01.g035150.t1.1	Zinc Finger (CCCH-Type) Family Protein	C3H	1.9
Cre10.g446900.t1.2	WD40 Repeat Proteinprl1/PRLl2-Related	Orphans	1.7
Cre02.g115250.t1.1	Centriole Proteome Protein	Orphans	1.6
Cre12.g523200.t1.1	Nucleosome Remodeling Factor	Orphans	1.6
Cre09.g389550.t1.1	Dnaj-Like Protein	MYB-related	1.6
Cre03.g197350.t1.2	Cell Division Cycle 5-Like Protein	MYB-related	1.5
Cre17.g713900.t1.2	Tor Kinase Binding Protein	Orphans	– ^*b*^
**Cre01.g020400.t1.2**	WD40 Repeat Protein	Orphans	2.3
**Cre09.g392350.t2.1**	Rna Recognition Motif (Rnp Domain) (Rrm_1)	CSD	1.9
**Cre01.g035000.t1.2**	Wd Repeat Protein	Orphans	1.8
**Cre17.g729150.t1.2**	Rna Recognition Motif. (Rnp Domain) (Rrm_1)	CSD	1.7
**Cre16.g662800.t1.2**	Splicing Factor, Component Of The U4/U6-U5 Snrnp Complex	Orphans	1.6
**Cre06.g275100.t1.2**	Splicing Factor 3B, Subunit 4	CSD	1.5
**Cre06.g274200.t1.2**	Histone H2A	CCAAT	– ^*b*^
**Cre12.g507650.t2.1**	Chloroplast Dnaj-Like Protein	MYB-related	– ^*b*^
**Transcription regulators**			
Cre16.g668200.t1.1	Chromatin Remodeling Protein, Contains Phd Zn-Finger	ARID	3.9
Cre16.g672300.t1.2	Swi/Snf-Related Chromatin Binding Protein	HMG	2.8
Cre01.g015050.t1.1	Unknown	SNF2	2.2
Cre07.g322450.t1.1	Pwwp Domain (Pwwp)//Set Domain (Set)	PHD	1.7
Cre08.g380151.t1.1	Phd-Finger (Phd)//Wstf, Hb1, Itc1P, Mbd9 Motif	PHD	1.7
Cre07.g334200.t1.2	Atp-Dependent Rna Helicase Ddx41-Related	SNF2	1.6
Cre02.g078700.t1.1	Lysine-Specific Demethylase 4A-Related	JUMONJI	1.5
Cre06.g261450.t1.2	Swi/Snf-Related Chromatin Binding Protein	HMG	– ^*b*^
**Cre17.g709550.t1.2**	Lysine-36 Demethylase/Jmjc Domain-Containing Histone Demethylase 1A	JUMONJI	1.8
**Cre01.g029450.t1.1**	Non-Histone Protein 10	HMG	1.5
**Cre08.g367300.t1.1**	Bromodomain Extra-Terminal - Bet	DDT	– ^*b*^
**Cre08.g358532.t1.1**	Gata Zinc Finger (Gata) // Bah Domain (Bah)	PHD	– ^*b*^

Only proteins with fold change ≥1.5 are shown in the table. Proteins more abundant in low CO_2_ conditions are indicated in bold.

^*a*^ The transcription factor (TF) family has been determined from the Plant transcription factor database.

^*b*^ Proteins have been detected only in one of the two conditions—low/high CO_2._

What also remains to be determined are the *cis*-DNA elements that respond to these TFs and TRs. *Chlamydomonas CAH1* is the only CCM gene for which regulatory elements have been systematically investigated and identified. The 5′ upstream 543 bp region of the *CAH1* gene was shown to contain a silencer region and an enhancer region with enhancer elements EE-1 (AGATTTTCACCGGTTGGAAGGAGGT) and EE-2 (CGACTTACGAA) (Kucho *et al.*, 1999, 2003). The upstream regions of duplicated genes, *CAH4* and *CAH5*, which confer CO_2_ dependence in the presence of light was narrowed to 194 bp. No similarities were seen between the upstream regions of *CAH4/5* and *CAH1*, and no shorter segments in this 194 bp have been identified as the elements responsible for the [CO_2_]-dependent transcription (Villand *et al.*, 1997). Potential genome-wide *cis*-DNA regulatory elements in *Chlamydomonas* that had been shifted from high to low [CO_2_] were identified by FAIRE-seq (Winck *et al.*, 2013). The potential regulatory regions in these genes require further validation.

While the above studies help identify specific transcription regulatory elements, *Chlamydomonas* also carries a post-transcriptional regulatory machinery of an extensive system of small RNAs (sRNAs), with three Argonaute and three Dicer-like proteins (Molnár *et al.*, 2007; Zhao *et al.*, 2007; Valli *et al.*, 2016; Chung *et al.*, 2019). This gene expression modulatory system with 6164 loci predicted to give rise to sRNAs (Muller *et al.*, 2020) requires identification of its potential targets. Whether this extensive system of sRNAs regulates the CCM requires investigation.

### Mechanisms of regulation of CCM induction: [CO_2_] is not the only cue

**Fig. 1.**
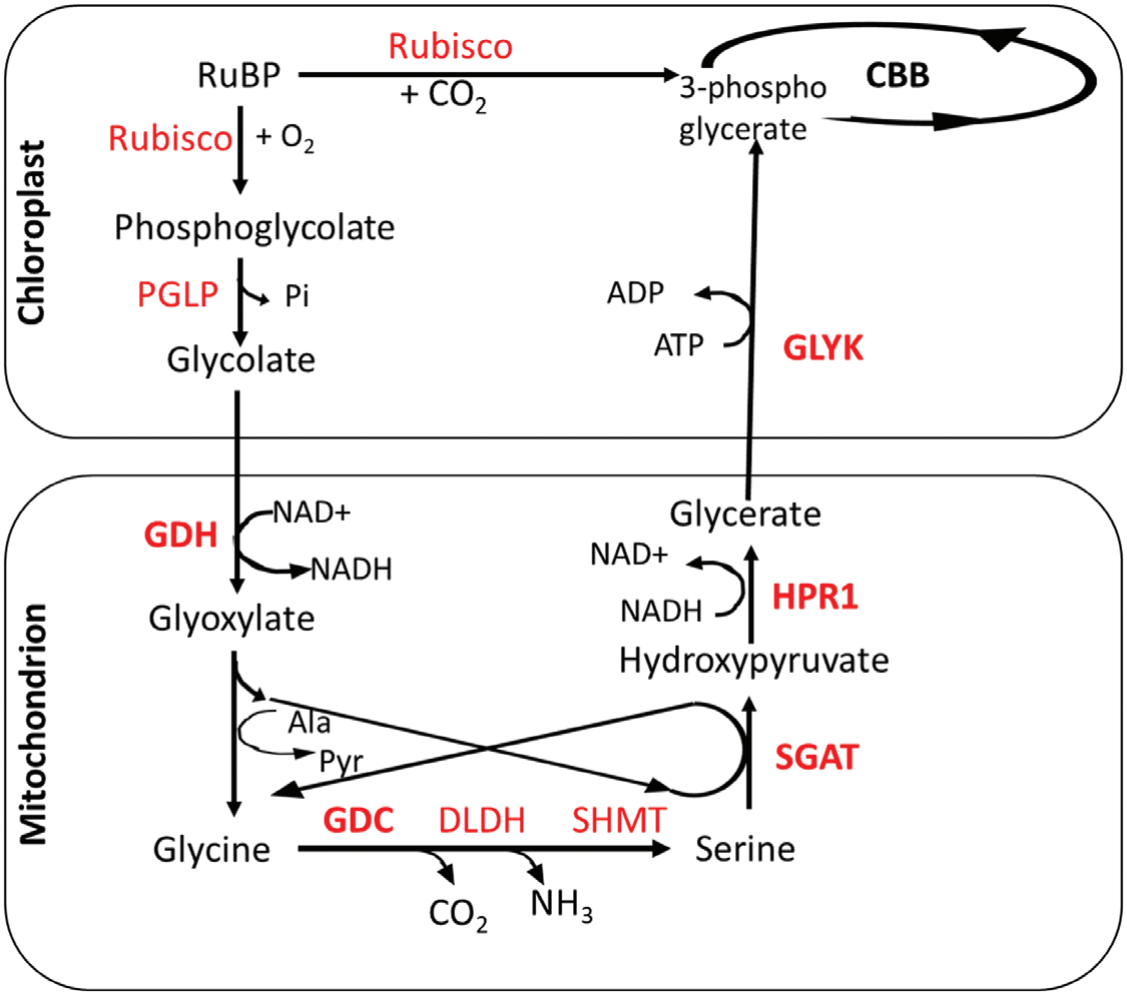
Photorespiratory cycle in *Chlamydomonas*. The enzymes Rubisco, PGLP (phosphoglycolate phosphatase), GDH (glycolate dehydrogenase), GGT (glutamate glyoxalate aminotransferase), GDC (glycine decarboxylase complex), SHMT (serine hydroxymethyl transferase), SGAT (serine/alanine glyoxalate aminotransferase), HPR1 (hydroxypyruvate reductase), and GLYK (glycerate kinase) are in red. Other abbreviations used: 2-OG, 2-oxoglutarate; Pyr, pyruvate. The enzymes highlighted in bold have expression dependent on both [CO_2_] and CIA5, similar to several CCM genes (Fang *et al.*, 2012).

**Fig. 2.**
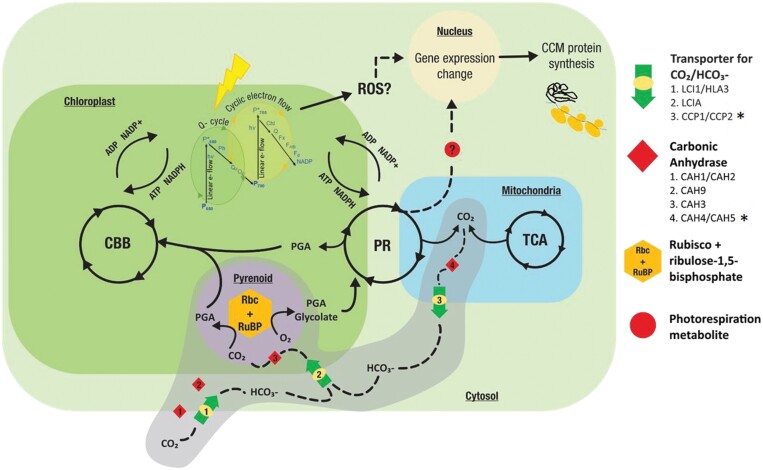
Schematic representation of crosstalk between photosynthetic electron transport (PET), the Calvin–Benson–Basham (CBB) cycle, photorespiration (PR), and the carbon concentration mechanism (CCM) in *Chlamydomonas.* CCM components: inorganic carbon transporters and carbonic anhydrases, occurring in various parts of the cell are highlighted in grey. *The role of mitochondrial proteins CCP1, CCP2, CAH4, and CAH5 is hypothesized, and remains to be explored. Reactive oxygen species (ROS) generated during PET, and PR metabolites are hypothesized to act as signalling molecules for the CCM.

The CCM genes that appear to be responsive to [CO_2_] changes may be responding to photosynthetic activity or carbohydrate metabolism as an indicator of Ci, and not directly to [CO_2_] changes. A process allied to photosynthetic carbon reduction that is of particular interest is photorespiration (PR). PR in photosynthetic organisms removes the toxic metabolite 2-phosphoglycolate (2-PG) arising from Rubisco’s oxygenase activity (Fig. 1; Table 2). A series of PR reactions occurring in chloroplasts and mitochondria regenerates the Calvin–Benson–Bassham (CBB) cycle intermediate 3-phosphoglycerate (3-PGA) from 2-PG, with the associated loss of 25% of fixed carbon as CO_2_. It must be noted that the *Chlamydomonas* PR differs from that in higher plants in two aspects: (i) glycolate is converted to glyoxylate in the mitochondria rather than the peroxisomes; and (ii) glyoxylate formation is catalysed by algal glycolate dehydrogenase (GDH), as opposed to glycolate oxidase. PR also acts as a sink for energy and reducing power from photosynthetic electron transfer (PET) when CBB cycle activity is limiting (Kozaki and Takeba, 1996; Moroney *et al.*, 2013). PR helps prevent blockades in reduced PET chains, which otherwise would result in formation of reactive oxygen species (ROS), with deleterious effects. ROS resulting from over-reduction of PET include ^1^O_2_ (singlet oxygen), H_2_O_2_ (hydrogen peroxide), O^−^_2_ (superoxide), and ·OH (hydroxyl radical). While ^1^O_2_ is generated predominantly at the reaction centre of PSII and is the main ROS responsible for photo-oxidative damage, the other three are formed at the acceptor side of PSI (Erickson *et al.*, 2015). ^1^O_2_ is formed primarily by energy transfer from the triplet state of photosensitizers such as chlorophyll, tetrapyrroles, and flavins. *Chlamydomonas* exhibits acclimation to ^1^O_2_ (Ledford *et al.*, 2007), and this acclimation response is an indicator of the ^1^O_2_ signalling mechanism (Erickson *et al.*, 2015). PR is thus intricately connected with photosynthesis and the CCM, and could help resolve imbalances in PET and the CBB cycle (Fig. 2) (Caspari *et al.*, 2017), by altering ROS levels. This interconnectedness suggests that metabolites resulting from PR and the CBB cycle, or ROS from light absorption by saturated PET chains, could be signals leading to CCM induction.

**Table 2.** List of photorespiration genes in *Chlamydomonas*

Gene ID	Short name	Brief description
*Cre03.g168700*	*PGLP1*	Phosphoglycolate phosphatase/4-nitrophenylphosphatase
*Cre10.g438100*	*PGLP2*	Phosphoglycolate phosphatase/4-nitrophenylphosphatase
*Cre03.g162601*	*PGLP3*	CDP-diacylglycerol-glycerol-3-phosphate 3-phosphatidyltransferase
***Cre06.g288700***	*GDH*	Glycolate dehydrogenase
***Cre10.g451950***	*AAT*	Alanine aminotransferase
*Cre06.g294650*	*AGT1*	Alanine-glyoxylate transaminase
*Cre03.g182800*	*AGT2*	Alanine-glyoxylate transaminase
***Cre12.g534800***	*GDC-P*	Glycine cleavage system, P protein
*Cre06.g253350*	*GDC-H*	Glycine cleavage system, H-protein
***Cre03.g193750***	*GDC-T*	Glycine cleavage system, T protein
*Cre18.g749847*	*DLDH*	Dihydrolipoyl dehydrogenase
*Cre16.g664550*	*SHMT1*	Serine hydroxymethyltransferase
*Cre06.g293950*	*SHMT2*	Serine hydroxymethyltransferase 2
*Cre09.g411900*	*SHMT3*	Serine hydroxymethyltransferase 3
***Cre01.g005150***	*SGAT*	Serine glyoxylate aminotransferase
***Cre06.g295450***	*HPR1*	Hydroxypyruvate reductase
***Cre12.g542300***	*GLYK*	Glycerate kinase

The genes highlighted in bold were identified to be regulated by both CIA5 and CO_2_, and were classified as having expression patterns similar to CCM clusters (Fang *et al.*, 2012)

The impact of PET rates was demonstrated by down-regulation of CCM genes *CAH1*, *HLA3*, *HLA1*, *HLA2*, and *HLA4* by 3-(3,4-dichlorophenyl)-1,1-dimethylurea (DCMU)—an inhibitor of PSII (Im and Grossman, 2002). DCMU also affects the intrachloroplastic localization of a retrograde signalling CCM protein called CAS, which in turn affects expression of 13 CCM genes including HLA3 and LCI1 (as discussed later). The use of DCMU leads to ^1^O_2_ accumulation (Fufezan *et al.*, 2002). Whether it is the downstream effects of ^1^O_2_ or the reduced photosynthesis that impacts gene expression requires further systematic investigations using modulators of photosynthesis and intracellular ROS. This study also showed that the above CCM genes require high-intensity light, in addition to low [CO_2_], to up-regulate their expression, again hinting towards PET or allied CBB cycle activity as potential influencers of CCM induction.

Like the CCM, PR activity is observed due to low [CO_2_]/[O_2_] in actively photosynthesizing organisms. Organisms with mutations of both PR and CCM genes have reduced ability to survive, unless in a high [CO_2_] environment (Moroney *et al.*, 2013). This led researchers to suggest a metabolite of PR as the signalling molecule for CCM induction in *Chlamydomonas* over three decades ago (Spalding *et al.*, 1985). A *Chlamydomonas* PR mutant lacking the phosphoglycolate phosphatase1 (PGP1) that converts 2-PG to glycolate, had affinity for inorganic carbon comparable with the wild type (WT) with an induced CCM (Suzuki *et al.*, 1990), leading the authors to suggest 2-PG as a CCM signalling molecule.

Further evidence for CCM–PR crosstalk comes from differential processing of glycolate in *Chlamydomonas* with or without a CCM (Moroney *et al.*, 1986). While glycolate is excreted under high CO_2_ conditions (CCM uninduced and PR down-regulated), glycolate excretion is minimal (1/80th of that in high CO_2_ conditions) in low CO_2_, CCM-induced conditions. Experiments with labelled CO_2_, labelled glycolate, and inhibitors of PR showed that this is not owing to lowered Rubisco oxygenase activity, but rather due to high rates of processing of glycolate by PR enzymes. This crosstalk between the algal CCM and PR (Fig. 1) is further strengthened by co-regulation of several CCM and PR genes by similar environmental stimuli of CO_2_ and the master regulator *CIA5* (Fang *et al.*, 2012). The PR genes are shown in Table 2, with those highlighted being co-expressed with CCM genes. Co-regulation of expression of PR and CCM genes is discussed further later in this review where we propose a hypothesis for evolution of CCM regulatory mechanisms. In addition to the transfer of metabolites between chloroplasts and mitochondria in *Chlamydomonas* during PR, light and CO_2_ induce changes in mitochondrial arrangement within the cells (Geraghty and Spalding, 1996; Polukhina *et al.*, 2016). The mitochondria lying between the chloroplast and cell membrane in CCM-active cells (Geraghty and Spalding, 1996) probably capture the glycolate exiting the chloroplast, and the implications for mitochondrial CAs and inorganic carbon transporters for the CCM are discussed in the last section. Whether the PR metabolites moving between organelles with light- and CO_2_-dependent intracellular location impact expression of nuclear CCM genes needs further investigation.

There have only been a few studies to explore the flux between PR, the CCM, and photosynthesis. Caspari *et al.* (2017) studied a pyrenoid-less CCM-defective mutant expressing a higher plant version of the Rubisco small subunit. This mutant exhibited a lower PET rate without affecting the intrapyrenoid thylakoid morphologies to compensate for the limited CO_2_ supply arising from lack of the CCM. The rate of PET was restored to levels comparable with those of WT cells when exposed to high CO_2_ and the mutant also had increased non-photochemical quenching (NPQ) to dissipate the energy reaching the photosystems.

A large-scale experiment studying metabolite flux and gene expression in photoautotrophic *Chlamydomonas* cells grown under low CO_2_ (0.04%) and high CO_2_ (10%) showed that the mitochondrial processes of glycolysis, gluconeogenesis, the glyoxylate pathway, dicarboxylate, and metabolism rates of amino acids in PR were responsive to [CO_2_] (Winck *et al.*, 2016). A more systematic analysis of PET, PR, and CBB cycle reactions and inorganic carbon uptake under different conditions of [CO_2_] is needed. Engineering of the biophysical (algal/cyanobacterial) CCM into higher plants might require tinkering with PR processes, in addition to introducing various CCM components (Atkinson *et al.*, 2016, 2020), for optimizing fluxes between light and dark reactions of photosynthesis.

While the CCM is active in the presence of light and the transcription of several of the CCM genes is light and [CO_2_] dependent (Brueggeman *et al.*, 2012; Fang *et al.*, 2012; Tirumani *et al.*, 2014), there have been observations of transcription of *CAH1*, *CAH3*, *CAH6*, and *LCIB* in low [CO_2_] conditions even in the dark (Rawat and Moroney, 1995; Mitchell *et al.*, 2014; Tirumani *et al.*, 2014, 2019). However, the increase of protein levels of these dark–low [CO_2_] transcribed genes, and localization of CAH3 to the pyrenoid, does not happen until the cells are exposed to light (Mitchell *et al.*, 2014; Tirumani *et al.*, 2014). These observations point towards regulation of expression not just during transcription, but also during translation and transport.

In conclusion, much ground remains to be covered for identification of key regulatory elements of the inducible CCM transcription machinery. Efforts are needed to utilize the extensive temporal transcription data from *Chlamydomonas* (Zones *et al.*, 2015; Strenkert *et al.*, 2019) to identify potential CCM TFs and TRs by developing gene regulatory networks (Emmert-Streib *et al.*, 2014). Experiments are needed to understand the CIA5 mechanism, and validate and characterize potential CCM TFs and TRs identified computationally or via organism-wide experiments discussed in this section. Understanding of CCM regulation requires a general comparison with other physiological mechanisms associated with sensing light and signalling photosynthetic, photorespiratory, and photoinhibitory responses. This interconnectedness of PR and CCM led us to consider their evolutionary origins in the section ‘Insights for evolution of CCM regulation’.

## Chaperones and the import and assembly of chloroplastic CCM proteins

Fewer than 100 genes encoding proteins are found in the chloroplast, with the remaining plastidic proteins, including those involved in the CCM, encoded in the nucleus (Martin *et al.*, 2002). The spatial segregation between nuclear gene expression and localization of the photosynthetic apparatus in the chloroplast means that many of the photosynthetic and CCM components need to be translated on cytoplasmic ribosomes and transported to/across the chloroplast membranes. This implies the need for chaperones for transport and folding of CCM proteins. In this section, we evaluate experimental evidence for chaperones and cellular transport machinery having roles in the assembly and functioning of the CCM.

### Folding and transport of CCM proteins

Based on existing data (Brueggeman *et al.*, 2012; Fang *et al.*, 2012; Wang *et al.*, 2015; Mackinder *et al.*, 2016) and proteomics studies, Mackinder *et al.* (2017) collated 624 nuclear genes involved in the CCM. Analysis of the encoded protein sequences suggests that a significant proportion are significantly disordered (140 sequences >70% disorder, 201 sequences >50% disorder, and 291 sequences >30% disorder). These included 76 chloroplast-localized CCM proteins of which 29, 21, and 16 sequences have disorderliness >30, 50, and 70%, respectively (Mackinder *et al.*, 2017). The occurrence of disorderliness is often an indicator of the presence of scaffolding modules for interaction with other proteins. Such disordered regions also destabilize proteins, and very often chaperones are required to prevent their irreversible misfolding (Pechmann and Frydman, 2014). Analysis of the 624 sequences also showed that 142 nuclear and 21 chloroplastic sequences carry at least one transmembrane (TM) domain (Krogh *et al.*, 2001). The insertion of TM regions into membranes requires the assistance of chaperones within the cell (Jarvis and Kessler, 2014; Guna and Hegde, 2018). The disorderliness and the presence of TM domains suggest a requirement for assisted folding in several of the CCM proteins that are translated on cytosolic ribosomes, as the proteins need to be transported across the chloroplast membranes (envelope and/or thylakoid) or embedded within them. Chloroplast TM transport is achieved through channels formed by TOC and TIC (translocon on the outer/inner chloroplast membrane) complexes. This transport is a complex process involving recognition of a transit peptide, protein unfolding and threading through TOC–TIC complexes, cleavage of the transit peptide in the stroma, and finally refolding of the protein. The chaperones Hsp70 (cytosolic and stromal), cytosolic Hsp90, stromal Hsp93, and Cpn60 are involved in chloroplast import of proteins (Flores-Pérez and Jarvis, 2013).

### Chaperones involved in the CCM

The roles of chaperones in organellar transport and folding mean that they could be vital for establishing the CCM, although little work has been carried out in this area. Here, using previously published protein interaction data, we have identified the CCM proteins that interact with chaperones HSP90, HSP70, and sHSPs (22E and 22F) (Table 3) (Mackinder *et al.*, 2017; Rütgers *et al.*, 2017). The list indicates that several key CCM proteins including EPYC1, Rubisco small subunits, Cas, and transporters might need chaperone assistance.

**Table 3.** CCM proteins that are found to interact with chaperones from proteomics studies (Mackinder *et al.*, 2017; Rutgers et al., 2017)

Protein ID	Description	Protein ID	Description
Interactors of HSP22C		Interactors of HSP70A (continued)	
Cre06.g295450	HRP1, hydroxypyruvate reductase	Cre03.g162800	LCI, low-CO2-inducible membrane protein
Cre05.g248450	CAH5, mitochondrial carbonic anhydrase	Cre16.g652800	Unannotated
Interactors of HSP22E		Cre04.g229300	RCA1, Rubisco activase
Cre10.g444700	SBE3, starch-binding enzyme	Cre12.g509050	PSBP3,OEE2-like protein of thylakoid lumen
Cre03.g151650	Unannotated	Cre13.g577100	ACP2, acyl-carrier protein
Cre16.g651050	CYC6, cytochrome *c*6	Cre16.g651050	CYC6, cytochrome *c*6
Interactors of HSP22F		Cre09.g394473	LCI9, low-CO_2_-inducible protein
Cre17.g724300	PSAK, PSI reaction centre subunit	Cre12.g560950	PSAG, PSI reaction centre subunit V
Cre16.g651050	CYC6, cytochrome *c*6	Cre10.g436550	EPYC1/LCI5, low-CO_2_-inducible protein
Cre12.g509050	PSBP3, OEE2-like protein of thylakoid lumen	Cre02.g120150	Unannotated
Cre10.g444700	SBE3, starch-binding enzyme	Cre02.g120100	RBCS1, Rubisco small subunit 1
Cre03.g179800	LCI24, low-CO_2_-inducible membrane protein	Cre07.g330250	PSAH, subunit H of PSI
Cre16.g663450	LCI11, low-CO_2_-inducible membrane protein	Cre14.g626700	PETF, apoferredoxin
Interactors of HSP70A		Cre12.g507300	LCI30, low-CO_2_-inducible protein
Cre16.g662600	Unannotated	Cre12.g519300	TEF9, unannotated
Cre10.g444700	SBE3, starch-binding enzyme	Interactors of HSP70B	
Cre06.g307500	LCIC, low-CO2 inducible protein	Cre16.g662600	Unannotated
Cre01.g054850	Unannotated	Cre16.g663450	LCI11, low-CO_2_-inducible membrane protein
Cre16.g663450	LCI11, low-CO_2_-inducible membrane protein	Cre06.g283750	HST1, homogentisate solanesyltransferase
Cre08.g372450	PSBQ, oxygen-evolving enhancer protein 3	Cre10.g444700	SBE3, starch-binding enzyme
Cre02.g097800	HLA3, ABC transporter	Cre03.g151650	Unannotated
Cre17.g724300	PSAK, PSI reaction centre subunit	Cre16.g652800	Unannotated
Cre09.g415700	CAH3, carbonic anhydrase 3	Cre17.g724300	PSAK, PSI reaction centre subunit
Cre04.g223300	CCP1, low-CO_2_-inducible chloroplast envelope protein	Cre09.g394473	LCI9, low-CO_2_-inducible protein
Cre10.g452800	LCIB, low-CO_2_-inducible protein	Cre06.g307500	LCIC, low-CO_2_-inducible protein
Cre05.g248450	CAH5, mitochondrial carbonic anhydrase	Cre06.g309000	NAR1.2, anion transporter
Cre06.g309000	NAR1.2, anion transporter	Cre12.g519300	TEF9, unannotated
Cre06.g295450	HRP1, putative hydroxypyruvate reductase	Cre03.g191250	LCI34, low-CO_2_-inducible protein
Cre08.g362900	PSBP4, lumenal PsbP-like protein	Cre01.g051500	ULP1, uncharacterized lumenal polypeptide
Cre03.g151650	Unannotated	Cre09.g415700	CAH3, carbonic anhydrase 3
Cre12.g485050	CAH6, carbonic anhydrase 6	Cre02.g097800	HLA3, ABC transporter
Cre01.g051500	ULP1, uncharacterized lumenal polypeptide	Cre10.g452800	LCIB, low-CO_2_-inducible protein
Cre03.g179800	LCI24, low-CO_2_-inducible membrane protein	Cre04.g229300	RCA1, Rubisco activase
Cre06.g283750	HST1, homogentisate solanesyltransferase	Cre12.g509050	PSBP3, OEE2-like protein of thylakoid lumen
Cre04.g223050	CAH2, carbonic anhydrase, alpha type, periplasmic	Cre02.g120100	RBCS1, Rubisco small subunit 1
Cre03.g191250	LCI34, low-CO_2_-inducible protein	Cre12.g560950	PSAG, PSI reaction centre subunit V
Interactors of HSP70B (continued)		Interactors of HSP90A	
Cre01.g054850	Unannotated	Cre02.g097800	HLA3, ABC transporter
Cre02.g120150	Unannotated	Cre04.g229300	RCA1, Rubisco activase
Cre03.g179800	LCI24, low-CO_2_-inducible membrane protein	Cre04.g223300	CCP1, low-CO_2_-inducible mitochondrial envelope protein
Cre04.g223300	CCP1, low-CO_2_-inducible chloroplast envelope protein	Cre07.g330250	PSAH, subunit H of PSI
Cre16.g651050	CYC6, cytochrome *c*6	Cre17.g724300	PSAK, PSI reaction centre subunit
Cre08.g372450	PSBQ, oxygen-evolving enhancer protein 3	Cre03.g151650	Unannotated
Cre10.g436550	EPYC1/LCI5, low-CO_2_-inducible protein	Cre12.g485050	CAH6, carbonic anhydrase 6
Cre08.g362900	PSBP4, lumenal PsbP-like protein	Cre16.g651050	CYC6, cytochrome *c*6
Cre04.g223050	CAH2, carbonic anhydrase, alpha type, periplasmic	Cre04.g223050	CAH2, carbonic anhydrase, alpha type, periplasmic
Cre12.g485050	CAH6, carbonic anhydrase 6	Cre09.g415700	CAH3, carbonic anhydrase 3
Cre03.g162800	LCI1, low-CO_2_-inducible membrane protein	Cre16.g662600	Unannotated
Cre05.g248450	CAH5, mitochondrial carbonic anhydrase	Cre01.g054850	Unannotated
Cre07.g330250	PSAH, subunit H of PSI	Cre16.g663450	LCI11, low-CO_2_-inducible membrane protein
Cre12.g507300	LCI30, low-CO_2_-inducible protein	Cre09.g394473	LCI9, low-CO_2_-inducible protein
Cre06.g295450	HRP1, putative hydroxypyruvate reductase	Cre10.g452800	LCIB, low-CO_2_-inducible protein
Cre14.g626700	PETF, apoferredoxin	Cre12.g507300	LCI30, low-CO_2_-inducible protein
Cre13.g577100	ACP2, acyl-carrier protein	Cre16.g652800	Unannotated
Cre17.g721500	STA2, granule-bound starch synthase I	Cre08.g362900	PSBP4, lumenal PsbP-like protein
Interactors of HSP70C		Interactors of HSP90B	
Cre05.g248450	CAH5, mitochondrial carbonic anhydrase	Cre07.g330250	PSAH, subunit H of PSI
Cre16.g662600	Unannotated	Cre03.g191250	LCI34, low-CO_2_-inducible protein
Cre10.g436550	EPYC1/LCI5, low-CO_2_-inducible protein	Cre12.g509050	PSBP3, OEE2-like protein of thylakoid lumen
Cre09.g394473	LCI9, low-CO_2_-inducible protein	Interactors of HSP90C	
Cre06.g295450	HRP1, putative hydroxypyruvate reductase	Cre10.g444700	SBE3, starch-binding enzyme
Cre06.g307500	LCIC, low-CO_2_-inducible protein	Cre17.g724300	PSAK, PSI reaction centre subunit
Cre16.g663450	LCI11, low-CO_2_-inducible membrane protein	Cre01.g054850	Unannotated
Cre03.g151650	Unannotated	Cre03.g151650	Unannotated
Cre06.g283750	HST1, homogentisate solanesyltransferase	Cre04.g229300	RCA1, Rubisco activase
Cre04.g229300	RCA1, Rubisco activase	Cre16.g663450	LCI11, low-CO_2_-inducible membrane protein
Cre16.g652800	Unannotated	Cre09.g415700	CAH3, carbonic anhydrase 3
Cre10.g444700	SBE3, starch-binding enzyme	Cre16.g652800	Unannotated
Cre06.g309000	NAR1.2m anion transporter		
Cre02.g097800	HLA3, ABC transporter		
Cre17.g724300	PSAK, PSI reaction centre subunit		
Cre16.g651050	CYC6, cytochrome *c*6		
Cre09.g415700	CAH3- carbonic anhydrase 3		
Cre04.g223300	CCP1, low-CO_2_-inducible mitochondrial envelope protein		

Despite the expectation that chaperones are essential for the CCM, the only chaperone which has been suggested to be essential for photosynthesis in a large-scale mutant screen is CDJ2, a chloroplastic DnaJ protein (Li *et al.*, 2019). Previously, expression of DNJ12 (DnaJ protein) was shown to be dependent on both CO_2_ and CIA5 (Fang *et al.*, 2012). Two other DnaJ chaperones, DNJ15 and DNJ31, have CIA5-dependent expression (Fang *et al.*, 2012), and hence feature as CCM proteins in a list compiled recently (Mackinder *et al.*, 2017). The expression of DNJ31 is also dependent on the retrograde signalling mediated by the CCM protein CAS (Wang *et al.*, 2016) (the role of CAS is discussed in ‘Retrograde signalling in CCM regulation’), further hinting at a role in the CCM. DnaJ or Hsp40 proteins are chaperones that work in conjunction with Hsp70 to help fold nascent proteins. Certain DnaJ proteins are known to confer substrate specificity. Whether the algal DnaJ proteins are indeed chaperones and are specifically interacting with CCM proteins needs further characterization. An up-regulation of HSF1 in response to low CO_2_ conditions in *Chlamydomonas* has also been shown (Winck *et al.*, 2013). HSF1 is a TF that is known to bind to promoter elements of HSP22F and HSP70A (Strenkert *et al.*, 2011), suggesting that CCM responses include an up-regulation of at least two chaperones. Overexpression of chaperones important for CCM expression might be worth considering in a strategy for engineering the CCM.

The limited evidence for chaperones associated with gene expression and protein import essential for the CCM should not negate their importance. Chaperones which are CCM specific might be few, and there might be several, such as Hsp70 and Hsp90 members, which cater for varied substrates, including CCM proteins. The varied nature of substrates for several chaperones makes it difficult to identify CCM-associated chaperones. The chaperones discussed in this section which have featured in genome-wide studies, and in interactomes, are good candidates to further explore this area.

## Dynamics of the starch sheath, pyrenoid matrix, and thylakoid tubules

While the response to extracellular environment changes rests primarily with the nuclear genes, the vital process of carbon capture occurs in the pyrenoids. The discovery of the role of EPYC1 as a linker protein (Mackinder *et al.*, 2016; Wunder *et al.*, 2018), the visualization of Rubisco–EPYC1 dynamics during pyrenoid division (Freeman Rosenzweig *et al.*, 2017), proteomics studies (Mackinder *et al.*, 2016, 2017; Zhan *et al.*, 2018), and the identification of a motif linking key elements (Meyer *et al.*, 2020) have given the CCM community much needed information about pyrenoidal composition and dynamics. In this section, we focus on specific aspects of the pyrenoid matrix, the extra-pyrenoidal starch sheath, the intra-pyrenoidal tubule network, as well as the linkages and interactions that promote their assembly.

### Role of the extra-pyrenoidal starch sheath

An extra-pyrenoidal starch sheath in *Chlamydomonas* was first clearly defined in 1957 (Sager and Palade, 1957). While the formation of a starch sheath under low [CO_2_] suggested that starch prevents CO_2_ leakage (Ramazanov *et al.*, 1994), there have been contradictory findings showing CCM induction in starchless algal mutants with a >10-fold increase in affinity for inorganic carbon (Plumed *et al.*, 1996; Villarejo *et al.*, 1996). However, recent studies in *Chlamydomonas* are indicative of a more fundamental role for the starch sheath in the CCM.

An important CCM protein associated with the starch sheath is LCIB that has low [CO_2_]- and light-dependent localization around the pyrenoid in a complex with LCIC. The localization of the LCIB–LCIC complex close to thylakoid tubule emergence and starch plate convergence (Yamano *et al.*, 2010, 2014), together with a preferential role in uptake of CO_2_ over HCO_3_^–^ during pH-dependent photosynthetic activity measurements (Wang and Spalding, 2014), are consistent with a role for the complex in capturing CO_2_ retro-diffusing from the pyrenoid. Whether the LCIB–LCIC complex acts as a physical barrier or functions as an inducible CA needs examination. Structures of both LCIB and LCIB–LCIC have attributes of CAs, but no detectable CA activity (Jin *et al.*, 2016). LCIB localization in extra-pyrenoidal starch affects pyrenoid size and number (Yamano *et al.*, 2014), and inorganic carbon affinity (Toyokawa *et al.*, 2020). LCID and LCIE are two more members of the same predicted CA family as LCIB occurring in *Chlamydomonas*, needing characterization (Wang and Spalding, 2014; Jin *et al.*, 2016).

The importance of starch in affecting pyrenoid number and orientation around the tubule network was further supported by studies with *Chlamydomonas saga1* mutants with lowered photosynthetic efficiency, and containing many pyrenoid-like structures, in contrast to the single pyrenoid in the WT (Itakura *et al.*, 2019). SAGA1 is believed to link starch and Rubisco. Pyrenoids harbour a thylakoid tubule network that acts as a conduit between thylakoid lumen and pyrenoid. In *saga1* mutants, while the number of pyrenoid-like structures is increased, there was only one tubule network which was often displaced to the periphery of a pyrenoid. Both formation of an entire starch sheath enclosing a single pyrenoid in the canonical position and a central thylakoid tubule network appear important for maintaining photosynthetic efficiency of the s*aga1* mutants. Recent findings show that SAGA2, 30% similar in sequence to SAGA1 with a starch-binding domain, localizes to the interface of the pyrenoid and starch sheath (Meyer *et al.*, 2020). Both SAGA1 and SAGA2 also contain a Rubisco-binding motif (RbM), the role of which is discussed later in this section.

### Thylakoid membrane proteins involved in the CCM

The function of the thylakoid tubule network is also thought to be critical for CCM induction. An important protein in the thylakoid lumen is the carbonic anhydrase CAH3, that becomes phosphorylated and localizes to the tubule network. Though interactions of CAH3 with thylakoid-associated kinases (Depège *et al.*, 2003; Lemeille *et al.*, 2010; Mackinder *et al.*, 2017) have been observed, the exact kinase responsible for its phosphorylation remains unknown. CAH3 is responsible for converting HCO_3_^–^ to CO_2_ for release into the heart of the pyrenoid (Blanco-Rivero *et al.*, 2012). Such a mechanism will require a HCO_3_^–^ transporter to be located in the thylakoid membrane, and *Chlamydomonas* cells lacking CAH3 grow more slowly compared with WT cells, particularly in low [CO_2_], highlighting its role in the upkeep of the CCM (Sinetova *et al.*, 2012). Recently, three genes coding for bestrophin-like proteins in *Chlamydomonas* called BST1–BST3 were identified (Mukherjee *et al.*, 2019). These genes are under the regulation of the CCM master TF CIA5 (Fang *et al.*, 2012), and interact with CCM components such as LCIB (Mackinder *et al.*, 2017). BST1–BST3 localize to the thylakoid membrane and are thought to transport HCO_3_^–^ ions to CAH3, with down-regulation of these proteins hampering cell growth at low [CO_2_] (Mukherjee *et al.*, 2019).

### Linkages involved in the formation of the pyrenoid matrix and starch sheath

Previous studies have shown that EPYC1, an intrinsically disordered repeat linker protein, is necessary to form a Rubisco–EPYC1 pyrenoidal matrix (Mackinder *et al.*, 2016). Recently, five Rubisco-binding regions in EPYC1 were identified using structural data for complexes of Rubisco and peptides representing different regions of EPYC1 (He *et al.*, 2020). EPYC1 acts as a ‘molecular glue’ that tethers multiple Rubisco molecules together to give rise to the pyrenoid matrix.

**Fig. 3.**
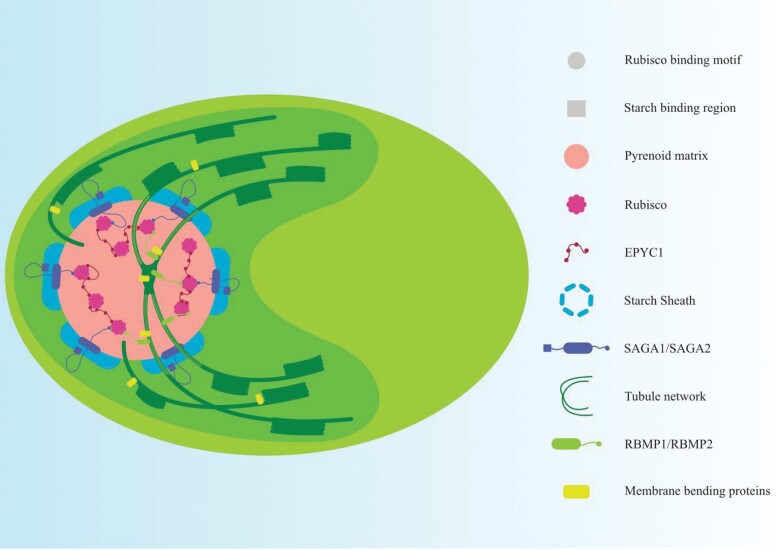
Assembly of the pyrenoid. The Rubisco-binding motif (RbM) mediates the formation of three regions of the pyrenoid. The RbM-bearing protein EPYC1 binds to multiple Rubisco holoenzymes and creates a Rubisco–EPYC1 condensate that forms the pyrenoid matrix. The interaction of RbM-bearing thylakoid-anchored proteins RBMP1 and RBMP2 with Rubisco tethers the pyrenoid matrix to the tubule network. The starch sheath is moulded around the pyrenoid matrix through the action of SAGA1 and SAGA2, which bind to Rubisco through their RbM domain and bind to the starch sheath through their starch-binding domain.

The presence of an RbM similar to that found in EPYC1 was identified in other pyrenoid-localized Rubisco-interacting proteins (Meyer *et al.*, 2020). Identified first in EPYC1, an RbM [D/N]W[R/K]XX[L/I/V/A] has been found in a putative chloroplast epimerase CSP41A, SAGA1, SAGA2, and in the thylakoid-localizing proteins RBMP1 and RBMP2. Disruption of the motif caused these pyrenoid-targeted proteins to diffuse homogeneously across the chloroplast, while introduction of the motif into a non-pyrenoidal protein led to their accumulation in the pyrenoid matrix (Meyer *et al.*, 2020). It must be noted that the presence of this motif does not always lead to pyrenoidal localization. As discussed previously, the presence of the motif in EPYC1 allows for its interaction with Rubisco to give rise to the EPYC1–Rubisco condensate forming the pyrenoid matrix (Wunder *et al.*, 2018; Atkinson *et al.*, 2020). Similarly, the presence of the RbM along with the starch-binding regions in SAGA2 might help the starch sheath envelop the pyrenoid. Two newly identified thylakoid tubule network-localizing proteins RBMP1(Cre06.g261750) and RBMP2 (Cre09.g416850) might help tether the pyrenoid to the tubules (Fig. 3). Among them, RBMP1 is predicted to be a member of the bestrophin family and may play a role in transporting HCO_3_^–^ to the pyrenoid directly through the tubule network, in contrast to the other bestrophin-like proteins which are found outside the pyrenoid. How these tubule network proteins are localized to the tubule network, which is formed even in cases where there is no pyrenoid matrix formation (Caspari *et al.*, 2017), needs investigation.

### Membrane bending and plasticity of the tubule network for CCM maintenance

A key area requiring study is the mechanism regulating the formation of the thylakoid tubule network for effective CCM operation. Insights for these processes may arise from studies of cell division in *Chlamydomonas*, when each of the four daughter cells normally contains a nascent pyrenoid as identified from fluorophore-tagged components (Freeman Rosenzweig *et al.*, 2017). Although a small proportion of cells synthesize a pyrenoid *de novo*, the extent to which the thylakoid tubule network is partitioned is not known. The intricate tubule network is formed as thylakoid membranes coalesce near entry points into the pyrenoid, but an added complexity arises from the internal mini-tubules which provide connectivity and allow CBB cycle intermediates to exchange between the pyrenoid matrix and the chloroplast stroma (Engel *et al.*, 2015). How this complex array of thylakoid membrane remodelling is regulated needs examination.

Membrane-remodelling proteins have been discovered in cyanobacteria and chloroplasts of algae and plants. However, there is limited understanding of their interactions during thylakoid tubule network formation and their potential to mediate CCM development. A set of proteins called CURT1 (Curvature Thylakoid 1) have emerged as important modulators of thylakoid membrane bending and plasticity. First identified in *Arabidopsis thaliana* (CURT1A, B, C, and D), these proteins are conserved across photosynthetic organisms, including three homologues in *Chlamydomonas* (Armbruster *et al.*, 2013). Arabidopsis CURT1 proteins concentrate around granal margins and oligomerize to induce membrane tubulation, with their inhibition negatively affecting photosynthetic efficiency (Armbruster *et al.*, 2013; Pribil *et al.*, 2018). The role of CURT in *Chlamydomonas* is pending investigation, but it probably contributes to similar membrane dynamics, and potentially plays a role in coordinating the assembly of CCM components, and the possible exclusion of PSII from thylakoid tubules within the pyrenoid matrix (McKay and Gibbs, 1991).

Another protein implicated in membrane remodelling is VIPP1, a member of the ESCRT-III family found in eukaryotes (Liu *et al.*, 2020, Preprint). Disrupting VIPP1 in vascular plants altered thylakoid structure and decreased the volume of thylakoid membranes, suggesting roles in thylakoid membrane upkeep, biogenesis, and remodelling (Kroll *et al.*, 2001; Westphal *et al.*, 2001; Hennig *et al.*, 2015, 2017; Heidrich *et al.*, 2017; Gutu *et al.*, 2018). VIPP1 localizes to the *Chlamydomonas* pyrenoid (Zhan *et al.*, 2018). The *vipp1* mutant is sensitive to high-light and heat stress, and shows altered thylakoid membrane structures close to the pyrenoid, suggesting a role in tubule biogenesis (Nordhues *et al.*, 2012). *Chlamydomonas* also harbours a paralogue of VIPP1, called VIPP2, that is not found in many land plants. VIPP1 and VIPP2 are both up-regulated under high-light- or H_2_O_2_-induced stress. Both oligomerize to form rod-like structures (Theis *et al.*, 2020). VIPP2 is expressed only in high-light conditions, in contrast to the constitutive expression of VIPP1, and forms a complex with VIPP1 and HSP22E/F. The lack of up-regulation of *HSP22E/F* in a *vipp2* mutant led the authors to suggest a role for VIPP2 in conveying chloroplastic stress to the nucleus. This recurring pattern of thylakoid membrane-remodelling proteins being up-regulated during high-light stress in *Chlamydomonas* is consistent with CCM-inductive stimuli (Im and Grossman, 2002). Notably, HSP22E/F interact with various starch synthesis proteins (Table 1), with the role of remobilization and starch plate formation being important for pyrenoid assembly and CCM induction (Ramazanov *et al.*, 1994; Itakura *et al.*, 2019). Another candidate involved in shaping thylakoid morphology is Fzl, which is a dynamin-like protein involved in grana organization in Arabidopsis (Gao *et al.*, 2006). A GTP-binding, oligomerizing homologue of this protein in *Chlamydomonas* called crFZL was recently found to be important for coping with high-light stress (Findinier *et al.*, 2019).

While the recent identifications of EPYC1 as a linker protein and of an RbM (Meyer *et al.*, 2020) are major developments, many intriguing questions related to thylakoid organization during CCM induction, whether during transfer from high to low CO_2_, or in synchronized cells during cell division and development, remain unanswered. Structural and mechanistic insights about the thylakoid membrane organizational proteins discussed in this section, in conjunction with life cycle and environmental regulation of the pyrenoid starch and tubule network, are needed to further our understanding of the CCM.

## Retrograde signalling in CCM regulation

In previous sections, we have navigated from the nucleus to the pyrenoid examining different algal mechanisms to establish and regulate the CCM. The chloroplast, effectively operating as the hub orchestrating photosynthetic reactions, must be sensitive to environmental factors that affect photosynthesis and/or the CCM. In this section, we see how changes in the chloroplast are communicated to the nucleus via signalling molecules (Rea *et al.*, 2018) for CCM regulation. In *Chlamydomonas*, molecular transducers such as tetrapyrrole intermediates, ROS, as well as Ca^2+^ ions help relay signals to the nucleus, with potential changes to the nuclear transcriptome. This section describes how changes within the chloroplast affect nuclear gene expression with implications for photosynthesis and the CCM.

### Redox status signalling in photosynthesis

Photosynthesis is a redox-centred metabolic process, subject to fluctuations in environmental conditions (Dietz *et al.*, 2016). Maintaining a functional PET requires balanced excitation of the two photosystems (Rea *et al.*, 2018) without which over-reduced PET components might generate harmful ROS. As discussed earlier, PET rates need to be tuned for the CCM, the CBB cycle, and PR (Caspari *et al.*, 2017). The influence of changes in PET affecting CCM gene expression (Im and Grossman, 2002), discussed above, suggests that the redox status of PET components acts as a regulator of CCM gene expression. It is therefore essential to have inter- and intraorganellar redox status communication (Pfannschmidt *et al.*, 2020) so that both the CCM and photosynthesis rates are optimal. Here, we explore the *gun4* mutant, which demonstrates a link between ROS generation and retrograde signalling.

### Retrograde signalling by GUN4

**Fig. 4.**
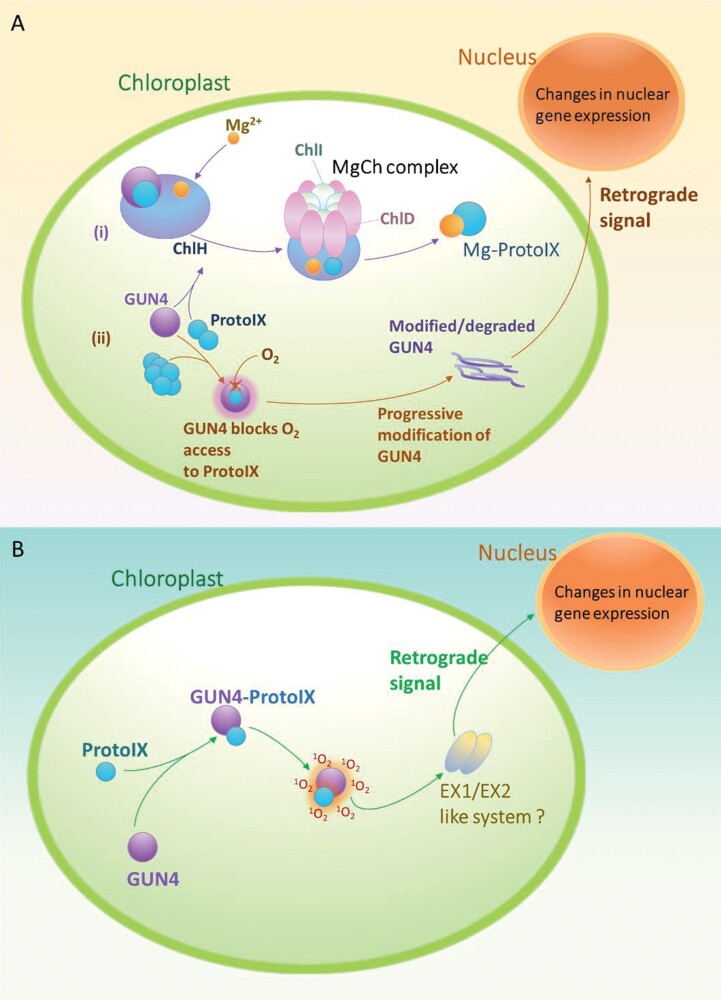
(A) GUN4 retrograde signalling model (after Brzezowski *et al.*, 2014). (i) GUN4 is proposed to be an activator of MgCh activity, interacting with the chlorophyll H subunit to promote the catalytic integration of Mg^2+^ with ProtoIX to form the chlorophyll biosynthesis pathway intermediate Mg-ProtoIX. (ii) The accumulation of excess tetrapyrrole intermediates, such as ProtoIX, in the chloroplast can lead to generation of ROS. GUN4 is proposed to bind ProtoIX, shielding its reaction with ROS. In shielding ProtoIX, GUN4 may be progressively modified or degraded, with degradation products hypothesized to act as the retrograde signals. (B) A contrasting model for GUN4 (after Tahari Tabrizi *et al.*, 2016). Instead of having a ‘shielding’ effect when bound to ProtoIX, the GUN4–ProtoIX complex appeared to escalate ^1^O_2_ generation. The elevated ^1^O_2_ produced by GUN4–ProtoIX may be sensed by an ^1^O_2_-sensing system (like the Arabidopsis EXECUTER1/EXECUTER2 or EX1/EX2 system) yet to be discovered, that relays a signal to the nucleus.

The biosynthesis of tetrapyrroles for chlorophyll generation is a process that is sensitive to oxidative stress and needs to be tightly regulated. Tetrapyrroles, as well as many of their biosynthetic intermediates, interact with oxygen in their triplet state to generate ROS, mainly singlet oxygen (Tanaka and Tanaka, 2007; Rea *et al.*, 2018). Tetrapyrrole metabolism occurs in the chloroplast, making use of nuclear-encoded enzymes, necessitating communication between the chloroplast and the nucleus. The role of tetrapyrrole intermediates in ‘retrograde signalling’ to modulate exyhpression of certain nuclear genes has been studied using ‘GUN mutants’ in plants and algae (Larkin, 2016). In WT cells, disrupted chlorophyll biosynthesis is known to activate a specific retrograde signal, which results in down-regulation of photosynthetic nuclear genes, such as *Lhcb2*. The *gun* mutant alleles are defective in this particular signal, instead elevating levels of these usually suppressed gene transcripts (Larkin, 2016). One such mutant—*gun4*—has been identified in *Chlamydomonas* with half as much chlorophyll as WT cells (Formighieri *et al.*, 2012). While GUN4 is not essential for chlorophyll synthesis, *gun4* mutants in Arabidopsis and *Chlamydomonas* exhibit impaired chlorophyll accumulation, suggesting a role for GUN4 in regulation of the enzyme Mg^2+^ chelatase (MgCh) (Fig. 4). RNA-seq analysis has shown that the expression of 803 nuclear-encoded genes in *Chlamydomonas* is altered in the *gun4* mutans as compared with the WT (Formighieri *et al.*, 2012).

While the role of retrograde signalling in regulating photosynthesis is accepted, we checked if it also plays a part in modulating the CCM. We compared the 803 differentially expressed genes in the *gun4* mutant (Formighieri *et al.*, 2012) and those identified as being important for the CCM (Mackinder *et al.*, 2017; Strenkert *et al.*, 2019). Our examination of these datasets shows that expression of nuclear genes encoding 52 CCM genes is affected >8-fold by GUN4 and, by extension, retrograde signalling (Table 4). This list of 52 genes includes a redox protein (peroxiredoxin), a PR pathway protein (serine hydroxymethyl transferase2), three photosystem components (*PSAK*, *PSAH*, and *PSIIPbs27*), bestrophin-3, three CAs (mitochondrial *CAH4* and *CAH5*, and periplasmic *CAH8*), and the chaperone *DNJ15.* That PR, mitochondrial CAs, and PET proteins are influenced by GUN4 further strengthens the crosstalk between these processes which has been highlighted in the section- ‘CCM induction and control of gene expression’. GUN4-influenced CCM genes also include *DNJ15*, implying protein homeostasis regulation by retrograde signalling. Seven of the CCM genes in Table 4 are uncharacterized. These proteins of unknown function which are influenced by GUN4 make a case for further exploration of the connections between the CCM and retrograde signalling.

**Table 4.** Nuclear-encoded CCM genes^*a*^ with >8-fold change in expression in the *gun4* mutant with respect to WT *Chlamydomonas*

Gene ID	Short name	Short description	Gene ID	Short name	Short description
*Cre01.g014350*	*PRX5*	Type II peroxiredoxin	***Cre08.g360200***	***DUR3***	**Urea active transporter**
*Cre01.g015350*		Light-dependent protochlorophyllide reductase	*Cre09.g405750*	*CAH8*	Carbonic anhydrase
***Cre01.g029250***		**Amino acid hydroxylase-like protein**	***Cre10.g426050***	***CTPA1***	**C-terminal processing peptidase**
*Cre01.g036950*		Cobalamin-5′-phosphate synthase	*Cre10.g455700*		Non-canonical poly(A) polymerase
*Cre01.g053950*	*MOX*	Monooxygenase	*Cre12.g485150*	*GAP1*	Glyceraldehyde 3-phosphate dehydrogenase
*Cre02.g078507*	*PSII Pbs27*	PSII Pbs27	*Cre12.g519300*	*TEF9*	Predicted protein
***Cre02.g085500***		**Putative transposase DNA-binding domain**	*Cre12.g535250*		RNA polymerase s factor
*Cre02.g107000*		Cyclin dependent kinase-2	*Cre12.g541550*		Unknown function
*Cre02.g143450*		Unknown function	***Cre12.g555700***	***DNJ15***	**DnaJ-like protein**
*Cre02.g144800*	*LCI8*	Acetylglutamate kinase	*Cre13.g569600*		Antibiotic biosynthesis monooxygenase
***Cre03.g149050***	***Cyt b561***	**Cytochrome *b*561**	*Cre14.g625450*		Methyltransferase
*Cre03.g158000*		Glu-1-semialdehyde aminotransferase	***Cre14.g630350***		**Unknown function**
*Cre03.g171350*	*SEC61A*	SEC61-α subunit	***Cre16.g652800***		**Unknown function**
*Cre03.g200350*		Methyltransferase	*Cre16.g658400*	*FDX2*	Ferredoxin
*Cre04.g223250*		LCIB-like gene	*Cre16.g659800*		Unknown function
***Cre05.g236650***	***CYG63***	**Guanylate cyclase**	***Cre16.g663450***	***BST-3***	**Bestrophin-3**
*Cre05.g248400*	*CAH4*	Mitochondrial carbonic anhydrase	***Cre16.g685100***		**Cobalamin synthesis protein**
*Cre05.g248450*	*CAH5*	Mitochondrial carbonic anhydrase	*Cre17.g700950*	*FDX5*	Apoferredoxin
*Cre06.g258850*		Stage V sporulation protein S	*Cre17.g713700*		Tryptophan pyrrolase
***Cre06.g266450***		**Protein kinase (MEC-15)**	***Cre17.g720900***		**Unknown function**
***Cre06.g284150***	***RHP2***	**Ammonium transport protein**	***Cre01.g038400***		**Calreticulin 2**
*Cre06.g303050*		Nitrate reductase	*Cre02.g111450*		Rhodanese-like protein
***Cre06.g310950***		**Sarcosine dehydrogenase**	*Cre03.g198950*		PSBP domain carrying protein
*Cre07.g315050*		Gamma-glutamyl hydrolase	*Cre06.g293950*	*SHMT2*	Serine hydroxymethyltransferase 2
*Cre07.g330250*	*PSAH*	PSI subunit H	*Cre07.g321400*	*FAP113*	Flagellar associated protein
***Cre07.g337100***		**Unknown function**	*Cre17.g724300*	*PSAK*	PSI subunit PsaK

All genes up-regulated in the *gun4* mutant are highlighted in bold.

^*a*^CCM genes were compiled from Mackinder *et al.* (2017) and Strenkert *et al.* (2019).

The mechanistic action of GUN4 in relaying a signal to the nucleus remains undetermined. GUN4 orthologues are present only in species that carry out oxygenic photosynthesis (Formighieri *et al.*, 2012), suggesting a role in photo-oxidative acclimation strategies. High resolution structures of Synechocystis GUN4 in the unliganded (Verdecia *et al.*, 2005) and protoporphyrin IX (ProtoIX) bound form (Chen *et al.*, 2015) have been solved. The binding pocket of GUN4 was shown to be amphiphilic and partially-open (Chen *et al.*, 2015). GUN4 that senses and binds the excess ProtoIX not entering chlorophyll synthesis, could be susceptible to modification by singlet oxygen (^1^O_2_). Modified and degraded GUN4 products are hypothesized to initiate the retrograde signalling to nucleus (Brzezowski *et al.*, 2014) (Fig 4a). Tahari Tabrizi *et al.*, suggest that the partially open binding pocket of GUN4 makes bound ProtoIX susceptible to photosensitization releasing ^1^O_2_. This ^1^O_2_ is hypothesized to relay signals through a system such as EXECUTER1 and EXECUTER2 (Tahari Tabrizi *et al.*, 2016) (Fig 4b), as seen in *Arabidopsis* (Singh *et al.*, 2015). It is proposed that a similar sensing system is required in *Chlamydomonas*. However, no corresponding homologue has been found; the most similarity shared with EXECUTER 1 and 2 was 10.2% and 11.2% by the *Chlamydomonas* protein Cre03.g163500. Mechanistic regulation of CCM gene expression by retrograde signalling in *Chlamydomonas*, as discussed in above for nuclear regulators, needs to be explored in detail with systematic analysis for signalling molecules, TFs, and *cis*-elements.

Another CCM protein that has been characterized in the last 5 years is CAS. It is the only CCM protein that has been studied in a systematic manner for its potential role in retrograde signalling independent of GUN4, and is described in the following paragraphs.

### Retrograde signalling by CAS

*Chlamydomonas* Ca^2+^-binding protein (CAS) is a chloroplastic thylakoid membrane protein that mediates signalling, as part of acclimation to high-light and low-carbon (LC) conditions (Wang *et al.*, 2016). CAS was initially studied in Arabidopsis, where it acts as a Ca^2+^-binding protein, regulating stomatal closure (Nomura and Shiina, 2014). Although no catalytic activity has been demonstrated for CAS, *A. thaliana* and *Chlamydomonas* CAS have Ca^2+^ binding ability in their N-terminus, and a rhodanese domain of unknown function (Wang, 2017). A comparison of the transcriptomes of a *cas* mutant strain with the WT and complemented strains demonstrated that absence of CAS leads to a >4-fold decrease in transcript levels of 13 genes (Wang *et al.*, 2016; Wang, 2017) (Table 5). All 13 genes except one coding for a predicted phosphatase have previously been identified as CCM genes. Of particular interest was reduced gene transcription and accumulation of transporters HLA3 and LCIA for the uptake of inorganic carbon into the cell (Yamano *et al.*, 2015) in the *cas* mutant. Three of the genes (*LCID*, *Cre12.g541550*, and *Cre26.g756747*) in Table 5 have been identified as CCM genes based on previous large-scale expression studies, but their cellular functions are unknown. The expression of CIA5-dependent *DNJ31*, encoding a DnaJ chaperone discussed above, is also influenced by CAS. These expression features of *DNJ31* make it an interesting candidate requiring its functional characterization and identification of its substrates. Two mitochondrial carbonic anhydrases (CAH4 and CAH5, also regulated by GUN4) and two mitochondrial envelope proteins (CCP1 and CCP2) of unknown function (Atkinson *et al.*, 2016; Mackinder *et al.*, 2017) are among those encoded by the 13 genes with CAS-dependent expression. The chloroplastic CCM protein CAS influencing the expression of four mitochondrial CCM proteins represents another feature of the mitochondria–chloroplast crosstalk needed to establish the CCM. Considering the importance of PR–CCM connections as described above, it is probable that CAH4 and CAH5 help recapture photorespired and respired CO_2_ in mitochondria as bicarbonate ions prior to export by unidentified transporters. CAS also influences PET by affecting expression of genes encoding two proteins involved in NPQ, namely LHCSR2 and LHCSR3. The over-reduction of the PET chain caused by high light intensity could be a potential trigger for CAS activation, and its modulation of NPQ (Caspari *et al.*, 2017). These observations further reiterate the need for tuning the rates of PET, PR, the CBB cycle, and inorganic carbon uptake with CCM induction, as discussed in above.

**Table 5.** Nuclear-encoded genes with upregulation >4-fold in WT *Chlamydomonas* with respect to the *cas* mutant

Protein id	Short name	Short description
*Cre02.g097800*	*HLA3*	ABC transporter
*Cre06.g309000*	*LCIA*	Anion transporter
*Cre03.g204577*	*DNJ31*	DnaJ-like protein
*Cre05.g248400*	*CAH4*	Mitochondrial carbonic anhydrase, beta type
*Cre05.g248450*	*CAH5*	Mitochondrial carbonic anhydrase
*Cre07.g334750* ^*a*^	*PPP30*	Protein phosphatase 2C
*Cre04.g223300*	*CCP1*	Low-CO_2_-inducible mitochondrial protein
*Cre04.g222750*	*CCP2*	Low-CO_2_-inducible mitochondrial protein
*Cre04.g222800*	*LCID*	Low-CO_2_-inducible protein
*Cre08.g367500*	*LHCSR3.1*	Stress-related chlorophyll *a*/*b* binding protein 2
*Cre08.g367400*	*LHCSR3.2*	Stress-related chlorophyll a/*b* binding protein 3
*Cre12.g541550*	*–*	Uncharacterized
*Cre26.g756747*	*–*	Uncharacterized

^*a*^ *PPP30* is an uncharacterized gene, which has not been identified as a CCM gene in any previous study.

**Fig. 5.**
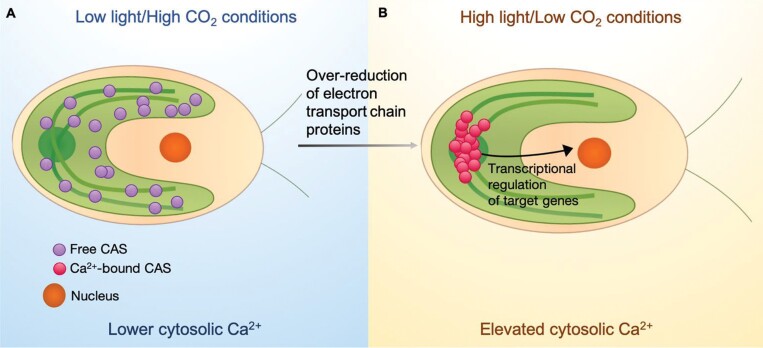
A schematic of a tentative mechanism for CAS activity in *Chlamydomonas* retrograde signalling. (A) Under low light/high CO_2_ conditions, CAS is dispersed throughout the chloroplast. (B) Under high light/low CO_2_, the ETC proteins become over-reduced, triggering the movement of CAS into the pyrenoid along the pyrenoid tubules. In the Ca^2+^-rich pyrenoid, CAS binds to Ca^2+^ and becomes activated. This form of CAS signals back to the nucleus to modulate target genes. It also induces an increase in intracellular Ca^2+^.

Similar to Rubisco, CAS was also revealed to move into the pyrenoid upon transition from high to low [CO_2_], with light being a prerequisite for this relocalization to occur (Yamano *et al.*, 2018). The importance of Ca^2+^ binding is demonstrated by use of the chelator BAPTA that lessens CAS-mediated accumulation of LCIA and HLA3 (Yamano *et al.*, 2018). The Ca^2+^-rich environment in the pyrenoid leads to Ca^2+^–CAS binding, which is thought to trigger a conformational change and mediate a signal to the nucleus, regulating CCM gene expression. The precise manner in which this protein signals for LC acclimation in *Chlamydomonas* is unknown, but a broad model is depicted in Fig. 5. The PET inhibitor DCMU prevents CAS relocalization under low [CO_2_] (Wang, 2017). Upon activation, CAS appears to relocate to a focal region within the pyrenoid. Though unclear, the signal propagation is affected by intracellular Ca^2+^ levels, [CO_2_], and light intensity. While the CAS mode of action is still unknown, these results suggest that the protein mediates a Ca^2+^-dependent retrograde signal to the nucleus, as part of the *Chlamydomonas* high light/LC acclimation. High light acclimation and CCM induction appear as important factors in the case of CIA5-mediated CCM gene expression and thylakoid membrane structuring proteins, and also in retrograde signalling in *Chlamydomonas*. CAS-mediated retrograde signalling further strengthens the ideas presented above, integrating the various physical and biological factors regulating CCM.

## Insights for evolution of CCM regulation

The cues regulating CCM genes, which have been frequently reiterated throughout this review, are not low ambient [CO_2_] alone, but also light intensity. The response to CO_2_ and light intensity changes, as discussed above, could be indirectly mediated by metabolites or ROS that result from photosynthesis and/or PR. The physiological connections between PR and the CCM along with the uncanny similarity in gene expression regulation of several CCM and PR genes, led us to hypothesize on the evolutionary origins of CCM regulatory mechanisms.

**Fig. 6.**
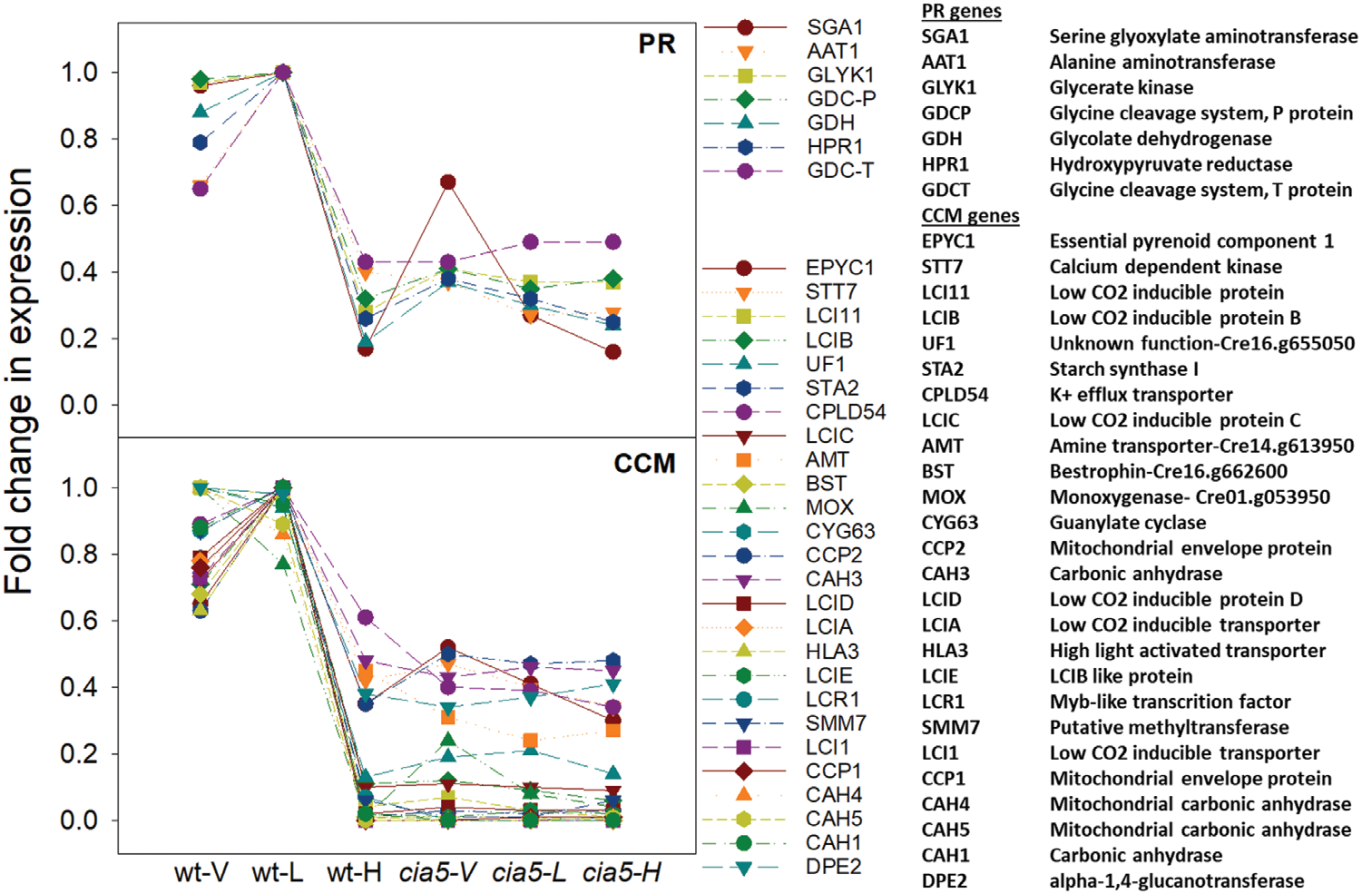
Relative expression of PR (top) and CCM (bottom) genes in wild-type (*wt*) and *cia5 Chlamydomonas* grown in different [CO_2_]: <0.02% (V, very low), 0.03–0.05% (L, low), and 5% (H, high). The expression of only genes classified as being in CCM clusters (Fang *et al.*, 2012) is shown here.

The regulation of PR in *Chlamydomonas* bears several similarities to that of the CCM. The genome-wide expression study with *cia5* showed that several key PR enzymes (Fig. 1; Table 2) [alanine aminotransferase1 (AAT1), glycerate kinase (GLYK), glycolate dehydrogenase (GDH), hydroxypyruvate reductase1(HPR1), serine glyoxylate aminotransferase1 (SGAT1), and glycine decarboxylase (GDC) complex enzymes] were dependent on the master regulator CIA5 and CO_2_ in a manner similar to several CCM genes (Fang *et al.*, 2012). The expression profiles of the PR and CCM genes that clustered in ‘CCM clusters’ (Fang *et al.*, 2012) are shown in Fig. 6. It is worth noting that not all PR genes display this expression pattern. Notable exceptions are *PGP* genes, serine hydroxymethyltransferase (*SHMT*) genes, and the *GDC-H* gene. GDC-H is in a GDC complex with two other subunits (GDC-T and GDC-P) that are encoded by genes which are part of ‘CCM clusters’. *PGP1* has already been discussed above. Although the PGP genes *PGP1*, *PGP2*, and *PGP3* did not show CIA5-dependent differential expression (Fang *et al.*, 2012), the expression of these PR genes was up-regulated under low [CO_2_] (Tural and Moroney, 2005). [CO_2_]-independent expression of *PGP* genes, in contrast to most genes that encode enzymes downstream of PGP in PR, lends more weight to the PGP substrate (2-PG) or product (glycolate) as CCM induction signalling molecules. 2-PG was suggested as a potential signalling molecule in a *pgp1* algal mutant study (Suzuki *et al.*, 1990). *SHMT* genes, which also have expression profiles different from that of other PR genes, encode proteins that catalyse conversion of glycine to serine. Whether the mitochondrial glycine:serine ratio impacts CCM induction requires investigation.

A recent study (Tirumani *et al.*, 2019) showed that genes encoding five of the PR enzymes, namely *AAT1*, *GDH*, *HPR1*, *PGP1*, and *GDC-H*, were not only dependent on low [CO_2_] for enhanced expression, but were also affected by light intensity. These genes also displayed diel regulation of expression without any circadian rhythmicity. These expression characteristics displayed by the five PR genes were also seen for four CCM genes (*CAH3*, *LCIB*, *LCI1*, and *CCP1*) in the same study. This similarity in expression profiles dependent on [CO_2_] changes and the presence of a common TF led us to look at the evolutionary origins of these processes. These studies provide compelling evidence that the stimulus for the CCM is likely to be determined by interactions between light intensity and PR activity when external CO_2_ is limiting.

The origin of oxygenic photosynthesis in cyanobacteria is placed ~2.4 billion years ago (bya) (Kopp *et al.*, 2005). The endosymbiotic event conferring eukaryotic cells with a photosynthetic chloroplast through engulfment of a cyanobacterium is believed to have occurred 1.8 bya, and the origin of chlorophytes (the branch carrying green algae such as *Chlamydomonas*) from a common ancestor for all green plants and algae is dated ~700 million years ago (mya) (Becker, 2013). The endosymbiotic events of internalizing cyanobacteria and proteobacteria are believed to have not only conveyed the photosynthetic machinery via chloroplast (cyanobacterial endosymbiosis) establishment, but also the PR enzymes from both cyano- and proteobacteria (mitochondrial evolution) (Eisenhut *et al.*, 2008; Bauwe *et al.*, 2012). PR had to develop in response to the oxygenase activity of Rubisco. PR is seen in all photosynthetic organisms, with and without biophysical (cyanobacteria, algae, and hornworts) or biochemical CCMs [C_4_ and Crassulacean acid metabolism (CAM) plants] (Hagemann *et al.*, 2016). It is an ancillary pathway to photosynthesis that evolved >1.8 bya, before the evolution of the algal CCM, which was perhaps as early as 500 mya, and C_4_ and CAM in land plants evolved <100 mya. The driver for evolution of PR, the biophysical CCM, C_4_, and CAM was the changing atmospheric composition with increasing [O_2_]:[CO_2_] ratios (Griffiths *et al.*, 2017). This similarity in evolutionary drivers and co-regulation by CIA5 of both PR and CCM led us to hypothesize that the cellular machinery adopted a pre-existing PR regulatory tool to establish the CCM.

The observation that Rubisco occurring in organisms with CCM/C_4_/CAM have greater affinity for O_2_ (i.e. a lower specificity factor) than those lacking concentration mechanisms (Griffiths *et al.*, 2017) hints at PR being an essential physiologically linked process for concentration mechanisms to function. Whether there are common TFs between PR and C_4_/CAM in higher plants, such as CIA5 of *Chlamydomonas*, requires further analysis. While common TFs such as CIA5 as a co-regulatory tool for concentration mechanisms and PR may not exist due to homoplastic origins of eukaryotic CCM, C_4_, and CAM pathways (Sage *et al.*, 2011; Sage, 2016; Raven *et al.*, 2017; Edwards, 2019), there is a possibility of evolution of other regulatory modes for coordinating the processes. However, the identification of common TFs regulating PR responses, as well as coordinating expression of the algal CCM and C_4_/CAM pathways in higher plants, is a promising line of investigation.

## Conclusions

We began this review by considering the role of the TF CIA5, and were faced with the confounding observation that this key regulatory molecule not only activates some CCM genes but is also involved in activating PR. Working from the hypothesis that [CO_2_] changes are sensed through indirect cues from photosynthetic activity or carbohydrate metabolism, we propose that signals associated with the imbalance of PET and CBB cycle rates in response to [CO_2_] and light intensity changes, such as photorespiratory metabolites, or associated redox signalling, mediated by specific chloroplastic retrograde signalling mediators such as CAS or GUN4, may be effectors in CCM induction. Whilst the CCM is a response to CO_2_ limitation, the associated signalling has been co-opted secondarily from the requirement to regulate genes processing photorespiratory intermediates, or cope with associated light stress when the PET is overenergized and NPQ is up-regulated (Caspari *et al.*, 2017). From an evolutionary perspective, this signalling would be consistent with the theory that CCM evolved in Chlorophyceae at the point of dissolved [CO_2_]:[O_2_] ratios being equivalent, some 400–500 mya (Griffiths *et al.*, 2017), probably building on the evolution of PR machinery that had evolved 1.8 bya (Becker, 2013). The close cooperation between mitochondria and chloroplast metabolite exchanges, PR activity, and inducible CCM components strengthens this notion. We also propose that the interconnections between PET, the CBB cycle, the CCM, and PR might be further strengthened by recapturing photorespired CO_2_ by the CCM machinery and suggest that optimization of an engineered algal CCM in higher plants requires consideration of the PR apparatus.

Whilst building on the tremendous progress that has been made in recent years in identifying novel CCM components and assembly of a functional pyrenoid (Meyer *et al.*, 2012, 2020; Mackinder *et al.*, 2016, 2017; Atkinson *et al.*, 2020), future studies should seek to identify the key effectors for CCM activation. The promise of identifying additional TFs (Arias *et al.*, 2020) and potential *cis*-activational motifs (Winck *et al.*, 2013), and post-transcriptional regulation through sRNA systems (Muller et al., 2020) will underpin efforts to understand CCM regulation. The analyses of large transcriptional datasets (Zones *et al.*, 2015; Strenkert *et al.*, 2019) using gene regulatory networks (Emmert-Streib *et al.*, 2014), in conjunction with *cis*-element analysis (Winck *et al.*, 2013), can help identify key TFs and TRs for future focus. Equally, by revisiting earlier studies which associated photorespiratory activity and CCM induction (Spalding *et al.*, 1985; Moroney *et al.*, 1986; Im and Grossman, 2002), we have highlighted additional regulatory processes which perhaps lead to CCM induction. Subsequent investigations with relevant knockout mutants from the large repositories generated (Li *et al.*, 2016, 2019; Vilarrasa-Blasi *et al.*, 2020, Preprint) will help test the above hypotheses and obtain further insights about regulation.

Major questions still remain, such as the origins and regulatory processes leading to the knotted thylakoid tubule network at the heart of the pyrenoid, the formation of associated connective minitubules, and spatial segregation between PSI and PSII. The energetic balance between CO_2_ reduction in the pyrenoid matrix, associated metabolite exchange, and regulation of the CBB cycle operating in the stroma may also account for the regulatory signalling complexities outlined above. The answers to questions about the complex regulatory processes leading to CCM induction will be revealed by molecular and physiological analyses and will require critical decisions on appropriate growth conditions (light intensity, photoperiods, synchronization of cells, and [CO_2_]) to identify the signalling mechanisms which orchestrate the biophysical CCM.
