# Correction to: Orchestral manoeuvres in the light: crosstalk needed for regulation of the *Chlamydomonas* carbon concentration mechanism

**DOI:** 10.1093/jxb/erac272

**Published:** 2022-07-21

**Authors:** Indu Santhanagopalan, Rachel Wong, Tanya Mathur, Howard Griffiths

**Affiliations:** Department of Plant Sciences, Downing Street, University of Cambridge, Cambridge CB2 3EA, UK; Department of Plant Sciences, Downing Street, University of Cambridge, Cambridge CB2 3EA, UK; Department of Plant Sciences, Downing Street, University of Cambridge, Cambridge CB2 3EA, UK; Department of Plant Sciences, Downing Street, University of Cambridge, Cambridge CB2 3EA, UK

Journal of Experimental Botany, Volume 72, Issue 13, 22 June 2021, Pages 4604–4624, https://doi.org/10.1093/jxb/erab169

During final editing of this manuscript, the authors regret that a key reference (Brzezowski *et al.*, 2014) was omitted. The authors are grateful for this opportunity to submit a correction, and for additional clarifications provided by Prof Bernhard Grimm.

Citations were added to the legend of Figure 4, and the paragraph commencing on page 4616 and ending on page 4617 was amended.

The version of record has been corrected directly.

New Figure 4 legend

Fig. 4. (A) GUN4 retrograde signalling model (after Brzezowski *et al*., 2014). (i) GUN4 is proposed to be an activator of MgCh activity, interacting with the chlorophyll H subunit to promote the catalytic integration of Mg^2+^ with ProtoIX to form the chlorophyll biosynthesis pathway intermediate Mg-ProtoIX. (ii) The accumulation of excess tetrapyrrole intermediates, such as ProtoIX, in the chloroplast can lead to generation of ROS. GUN4 is proposed to bind ProtoIX, shielding its reaction with ROS. In shielding ProtoIX, GUN4 may be progressively modified or degraded, with degradation products hypothesized to act as the retrograde signals. (B) A contrasting model for GUN4 (after Tahari Tabrizi *et al*., 2016). Instead of having a ‘shielding’ effect when bound to ProtoIX, the GUN4–ProtoIX complex appeared to escalate ^1^O_2_ generation. The elevated ^1^O_2_ produced by GUN4–ProtoIX may be sensed by an ^1^O_2_-sensing system (like the Arabidopsis EXECUTER1/EXECUTER2 or EX1/EX2 system) yet to be discovered, that relays a signal to the nucleus.

New paragraph (pp. 4616–4617)

The mechanistic action of GUN4 in relaying a signal to the nucleus remains undetermined. GUN4 orthologues are present only in species that carry out oxygenic photosynthesis (Formighieri *et al*., 2012), suggesting a role in photo-oxidative acclimation strategies. High resolution structures of *Synechocystis* GUN4 in the unliganded (Verdecia *et al*., 2005) and protoporphyrin IX (ProtoIX) bound form (Chen *et al*., 2015) have been solved. The binding pocket of GUN4 was shown to be amphiphilic and partially open (Chen *et al*., 2015). GUN4 that senses and binds the excess ProtoIX not entering chlorophyll synthesis could be susceptible to modification by singlet oxygen (^1^O_2_). Modified and degraded GUN4 products are hypothesized to initiate the retrograde signalling to nucleus (Brzezowski *et al*., 2014) (Fig. 4a). Tahari Tabrizi *et al*. suggest that the partially open binding pocket of GUN4 makes bound ProtoIX susceptible to photosensitization releasing ^1^O_2_. This ^1^O_2_ is hypothesized to relay signals through a system such as EXECUTER1 and EXECUTER2 (Tahari Tabrizi *et al*., 2016) (Fig. 4b), as seen in Arabidopsis (Singh *et al*., 2015). It is proposed that a similar sensing system is required in *Chlamydomonas*. However, no corresponding homologue has been found; the most similarity shared with EXECUTER 1 and 2 was 10.2% and 11.2% by the *Chlamydomonas* protein Cre03.g163500. Mechanistic regulation of CCM gene expression by retrograde signalling in *Chlamydomonas*, as discussed in above for nuclear regulators, needs to be explored in detail with systematic analysis for signalling molecules, TFs, and *cis*-elements.

